# Evaluating the therapeutic benefit of bacterial lysates in chronic obstructive pulmonary disease: a systematic review and meta-analysis

**DOI:** 10.3389/fmed.2026.1928291

**Published:** 2026-10-21

**Authors:** Azizah Almujadib, Reham Alotaibi, Aseel Alsultan, Reem Alshehri, Haneen H. Alzahrani, Noor Almahfoodh, Bassam Asiri, Shaden Alsulami, Shahad Alghool, Ghidi Saddiqi, John R. Hurst, Hajed M. Alotaibi, Malik A. Althobiani

**Affiliations:** 1 College of Pharmacy, King Khalid University, Abha, Saudi Arabia; 2 Respiratory Therapy, Almaarefa University, Riyadh, Saudi Arabia; 3 Respiratory Therapy, Princess Nourah Bint Abdulrahman University, Riyadh, Saudi Arabia; 4 College of Pharmacy, King Khalid University, Abha, Saudi Arabia; 5 College of Medicine, King Khalid University, Abha, Saudi Arabia; 6 College of Medicine, Imam Abdulrahman Bin Faisal University, Dammam, Saudi Arabia; 7 College of Medicine, King Saud bin Abdulaziz University for Health Sciences, Riyadh, Saudi Arabia; 8 College of Medicine, King Abdulaziz University, Jeddah, Saudi Arabia; 9 College of Medicine, Umm Al-Qura University, Makkah, Saudi Arabia; 10 UCL Respiratory, University College London, London, United Kingdom; 11 Department of Respiratory Therapy, Faculty of Medical Rehabilitation Sciences, King Abdulaziz University, Jeddah, Saudi Arabia; 12 Respiratory Therapy Unit, King Abdulaziz University Hospital, King Abdulaziz University, Jeddah, Saudi Arabia

**Keywords:** alkaline bacterial lysates, Bacterial lysates, COPD, Ismigen, OM-85

## Abstract

**Background:**

Chronic obstructive pulmonary disease (COPD) with exacerbations often triggered by infections. Bacterial lysates have been investigated as interventions to reduce the exacerbations of COPD.

**Methods:**

We conducted a systematic review and meta-analysis of randomized controlled trials (RCTs) evaluating bacterial lysates (BLs) in adults with chronic obstructive pulmonary disease (COPD). PubMed, EMBASE, CENTRAL, Web of Science, Google Scholar, ICTRP, and ClinicalTrials.gov were searched. In adults with COPD that compared bacterial lysates with placebo or conventional COPD therapy. Primary outcomes were the effect of BLs on exacerbations and duration. Secondary outcomes were hospitalisation risk, the mean number of exacerbations per participant during trial follow up, symptom scores for dyspnoea, sputum, cough, and adverse events. We used random-effects meta-analysis, assessed risk of bias with Cochrane RoB 2, graded certainty with GRADE.

**Results:**

Fifteen RCTs were included. The pooled risk ratio (RR) for exacerbation in patients with COPD treated with BLs vs. control was 0.83 (95% CI 0.74–0.93; *P* = 0.0009). The mean difference (MD) for exacerbation duration showed no statistical significant reduction −9.26 Days (95% CI −19.56 to 1.03; *P* = 0.08). Mean number of exacerbations (MD) −0.19 (95% CI −0.30 to −0.08; *P* = 0.0009). Hospitalisation RR was 0.52 (95% CI 0.35–0.78; *P* = 0.001).Symptom outcomes were dyspnoea standardised mean difference (SMD) −0.42 (95% CI −0.75 to −0.10; *P* = 0.01), cough (SMD) −0.37 (95% CI −0.63 to −0.11; *P* = 0.006), while sputum production showed no significant change (SMD) −0.20 (95% CI −0.65 to 0.25; *P* = 0.39), The RR for adverse events was 0.95 (95% CI 0.86–1.04; *P* = 0.27).

**Conclusion:**

Bacterial lysates may be associated with lower COPD exacerbation and hospitalisation risk, with small numerical improvements in dyspnoea, cough, sputum production showed no significant improvement; effects varied by formulation, these findings require confirmation in formulation specific trials.

**Systematic Review registration:**

https://www.crd.york.ac.uk/PROSPERO/view/CRD42024570783, PROSPERO CRD42024570783.

## 1 Introduction

Chronic obstructive pulmonary disease is a major global health problem with substantial morbidity and mortality (1, 2). It is currently ranked as the third leading cause of death globally, and in high-income settings is mostly caused by cigarette smoking. COPD involves persistent airflow obstruction, often with incomplete reversibility, due to chronic bronchitis, small-airways disease, emphysema, or a combination of these (1, 3). COPD exacerbations are commonly triggered by respiratory viral or bacterial infections, and episodes can also be precipitated by increased air pollution (4–6). Multiple factors contribute to exacerbation risk (7, 8). The bacteria implicated in exacerbations are often common colonisers of the respiratory tract, such as Haemophilus influenzae, Streptococcus pneumoniae, and Moraxella catarrhalis (9). COPD treatments currently available have been shown to reduce symptoms and exacerbations while improving lung function, exercise capacity, and overall health-related quality of life. In addition to established anti-inflammatory agents and bronchodilators (9), our systemic review and meta-analysis examine emerging drug therapy that may modulate immune responses in COPD, highlighting their potential to expand current treatment strategies. These include bacterial lysates (BLs), which have been investigated as a preventive strategy for reducing COPD exacerbations. BLs are generated by lysing respiratory-pathogen strains and bacterial components, enabling these fragments to interact with the host immune system (9). Several bacterial lysate formulations have been investigated as immunomodulatory therapies for preventing recurrent respiratory infections and COPD exacerbations. These preparations differ according to their manufacturing process and route of administration. Polyvalent mechanical bacterial lysates (PMBLs), such as Ismigen, are freeze-dried BLs derived from eight common respiratory pathogens that preserve bacterial surface antigens through mechanical disruption and are administered sublingually to stimulate regional mucosal immunity (10, 11). On the other hand, alkaline bacterial lysates, including OM-85, are produced using chemical lysis and are administered orally to activate the gut–lung immune axis (12). Another formulation, Diribiotine CK, consists of lysates derived from Klebsiella pneumoniae and Escherichia coli, although details regarding its manufacturing process have not been reported. Bacterial lysates have been evaluated in several randomized controlled trials, reported findings remain inconsistent owing to differences in bacterial lysate formulations, patient populations, disease severity, or didn’t assess Certainty of evidence using GRADE.

This systematic review and meta-analysis aims to evaluate the effectiveness of BLs in reducing exacerbation risk and hospitalisation in adults with COPD, compared with placebo or standard care. We also conducted exploratory analyses to assess whether treatment effects varied according to bacterial lysate formulation and disease severity. Based on evidence from randomised controlled trials. The study was registered at PROSPERO (registration number: CRD42024570783) and followed the Preferred Reporting Items for Systematic Review and Meta-Analysis (PRISMA) guidelines (13).

### 1.1 Search strategy

We searched PubMed, EMBASE, Google Scholar, Web of Science, CENTRAL, ICTRP, and ClinicalTrials.gov, using relevant keywords and synonyms to identify randomised controlled trials published before January 2025. This search was updated in September 2025 as the final database search and further enhanced by manual search. The inclusion criteria, based on the PICO model, focused on adults aged 18 years and older diagnosed with COPD. The interventions considered were the administration of BLs such as OM-85 (alkaline BL), Ismigen (PMBL) and Diribiotine CK (The manufacturing method was not reported). These were compared with either a placebo or conventional COPD therapy. The risk and duration of acute exacerbations were the primary outcome, while the mean number of exacerbations per participant during trial follow up, hospitalisation, and improvements in symptoms such as dyspnoea, sputum production, and cough were considered as secondary outcomes. Safety outcomes focused on adverse events. Only human RCTs, regardless of language. Studies that failed to meet the inclusion criteria were excluded.

The complete search strategy, including Boolean operators and MeSH terms, is detailed in Supplementary Table.

### 1.2 Study selection, data extraction, and quality assessment

Two reviewers independently screened studies and assessed full texts (14), with disagreements resolved by a third reviewer. Data were extracted and cross-checked by six reviewers, with missing information requested from authors when necessary. Two reviewers independently verified the data and assessed the risk of bias using the Cochrane RoB 2 tool for RCTs (13, 15–17).

### 1.3 Statistical analysis

Meta-analysis was performed using a random-effects model in Review Manager (version 5.4.1) (18). Model selection was based on the anticipated clinical and methodological heterogeneity rather than solely on the magnitude of *I*^2^. Continuous outcomes were analysed as mean difference (MD) or standardized mean difference (SMD), and dichotomous outcomes as risk ratio (RR) or odds ratio (OR), all with 95% confidence intervals (CIs). Heterogeneity was assessed using the chi-square test and *I*^2^ statistic (19). Prespecified subgroup and sensitivity analyses were conducted, and the certainty of evidence was evaluated using the GRADE approach.

## 2 Results

The database search identified 3,883 records, of which 15 studies (1985–2020) met the inclusion criteria Figure 1. Six studies evaluated mechanical bacterial lysates, eight assessed alkaline bacterial lysates, and one investigated diribiotine CK. Study characteristics are summarized in Supplementary Table 1, and participant demographics are presented in Supplementary Table 2.

**Figure 1:**
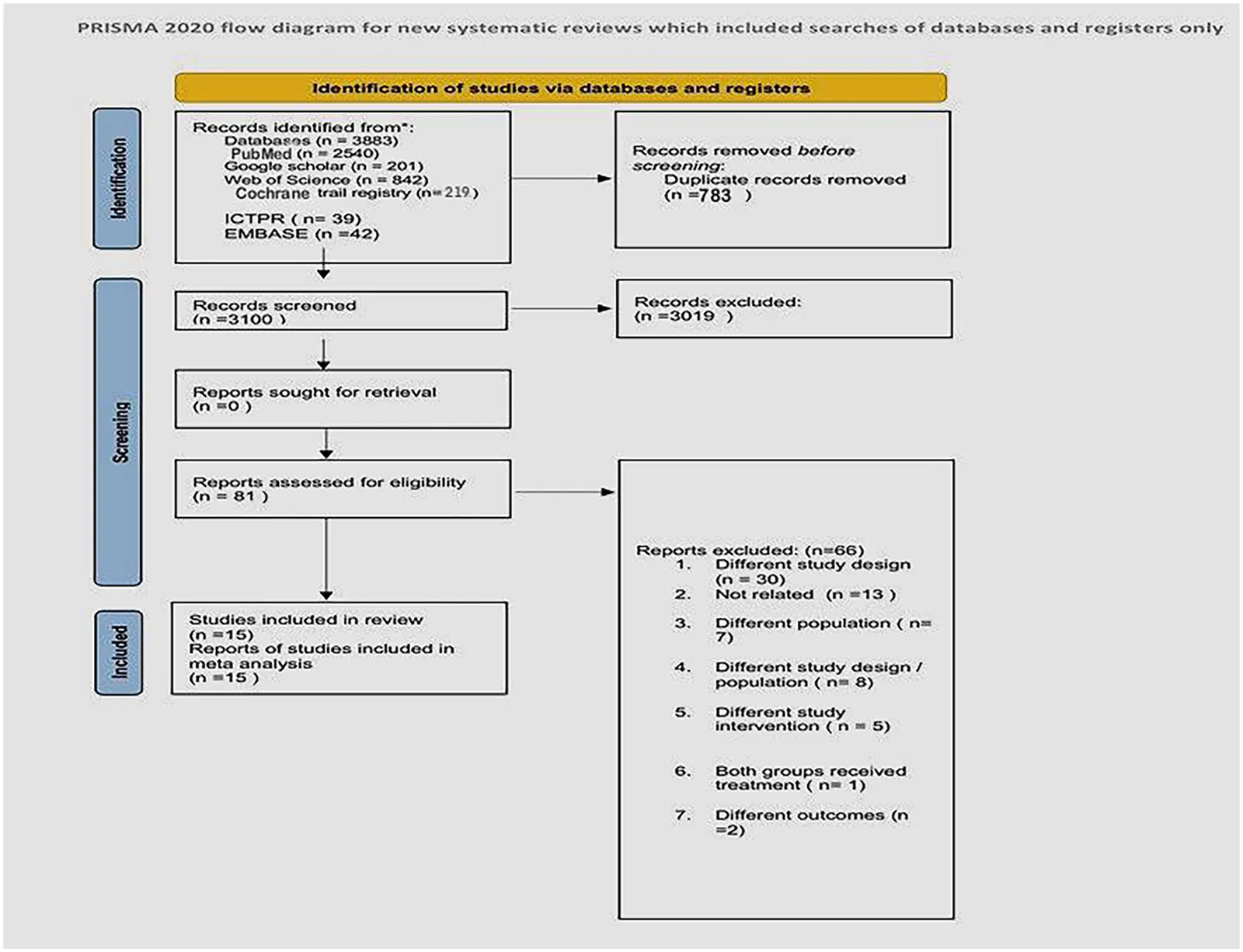
PRISMA diagram summarises the study selection process.

### 2.1 Risk of bias analysis

Quality of Included Studies (Risk of Bias).

Figures 2, 3 summarises the risk of bias assessment (16, 17).

**Figure 2:**
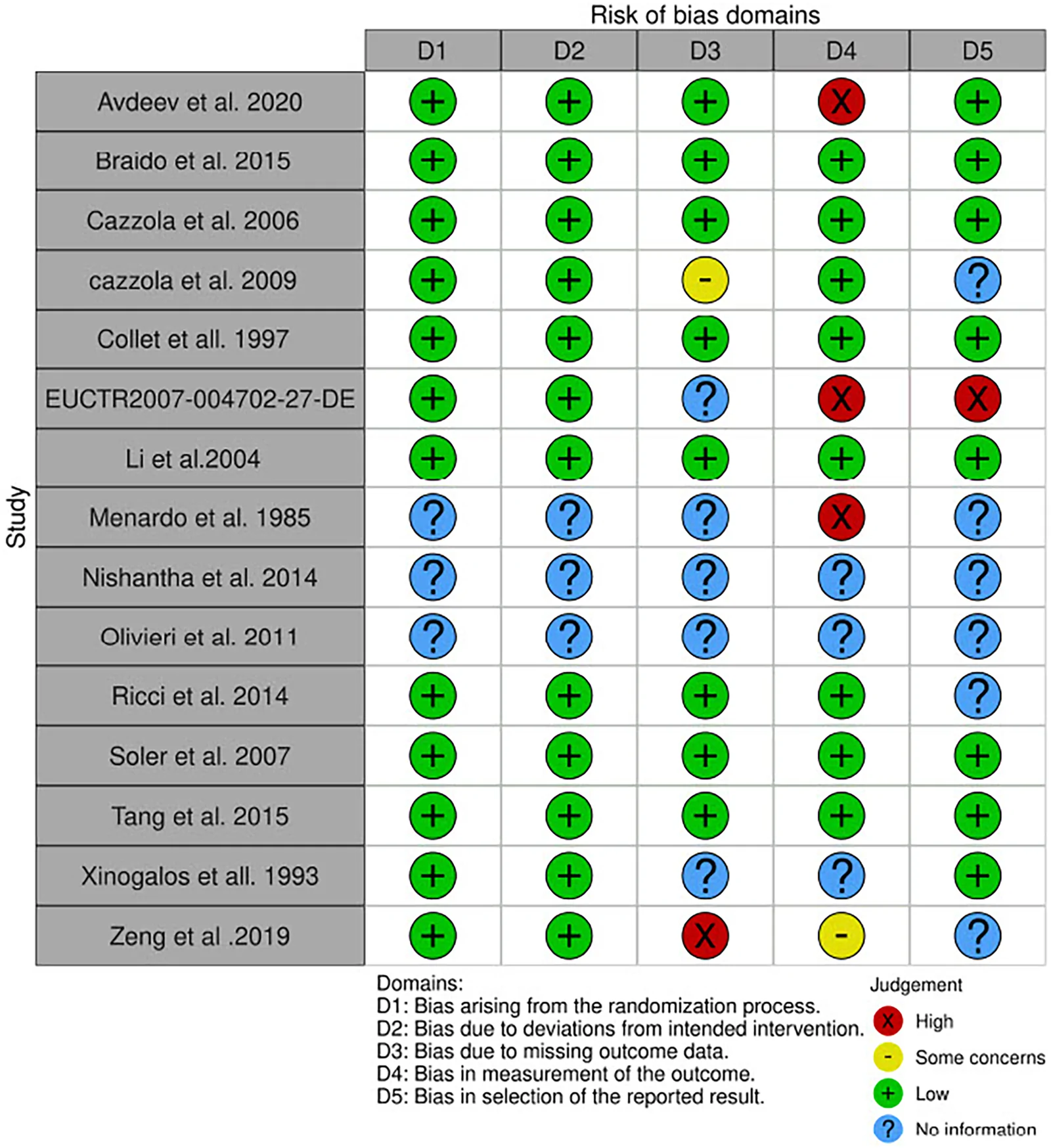
Risk of bias item for each included study (9, 20–31).

**Figure 3:**
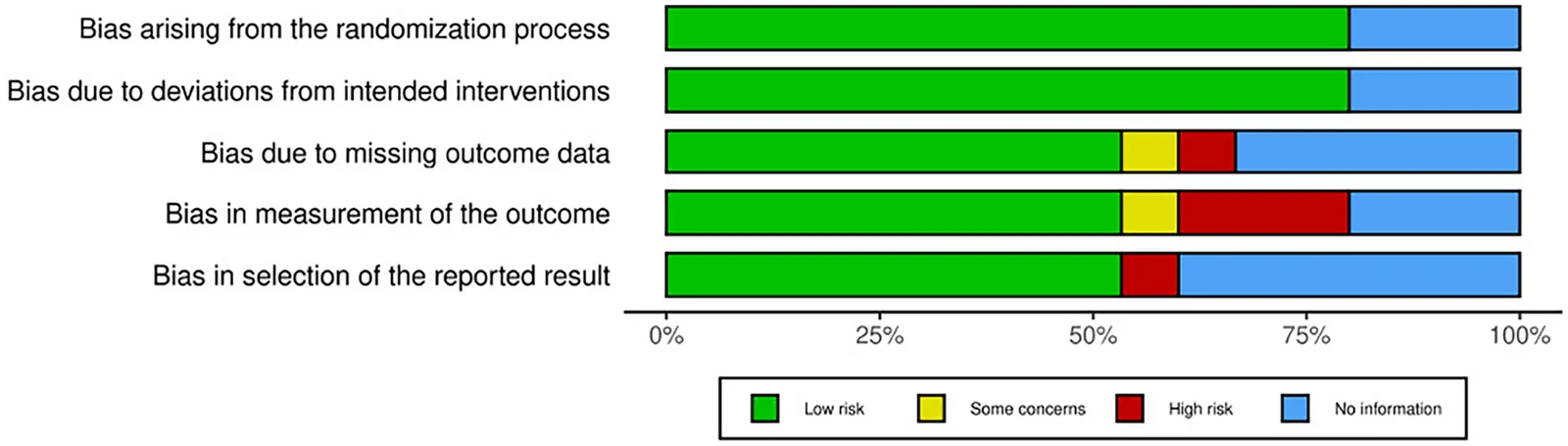
Detailed summary table and traffic light plots of the risk of bias assessment.

### 2.2 The effect of using Bl on exacerbation

Ten RCTs contributed to the pooled analysis of exacerbation risk. Bacterial lysates significantly reduced the risk of COPD exacerbations compared with control RR 0.83 (95% CI 0.74 to 0.93; *P* = 0.0009). With moderate heterogeneity *I*^2^ = 51%. Corresponding to an approximate 17% relative reduction. Subgroup analyses suggested that this benefit was primarily driven by alkaline bacterial lysates, whereas evidence for PMBLs remained inconclusive because of substantial heterogeneity and limited sample size. Figure 4 presents the forest plot of the effect of using BL on exacerbation (9, 11, 12, 20, 23, 25, 28, 30–32).

**Figure 4:**
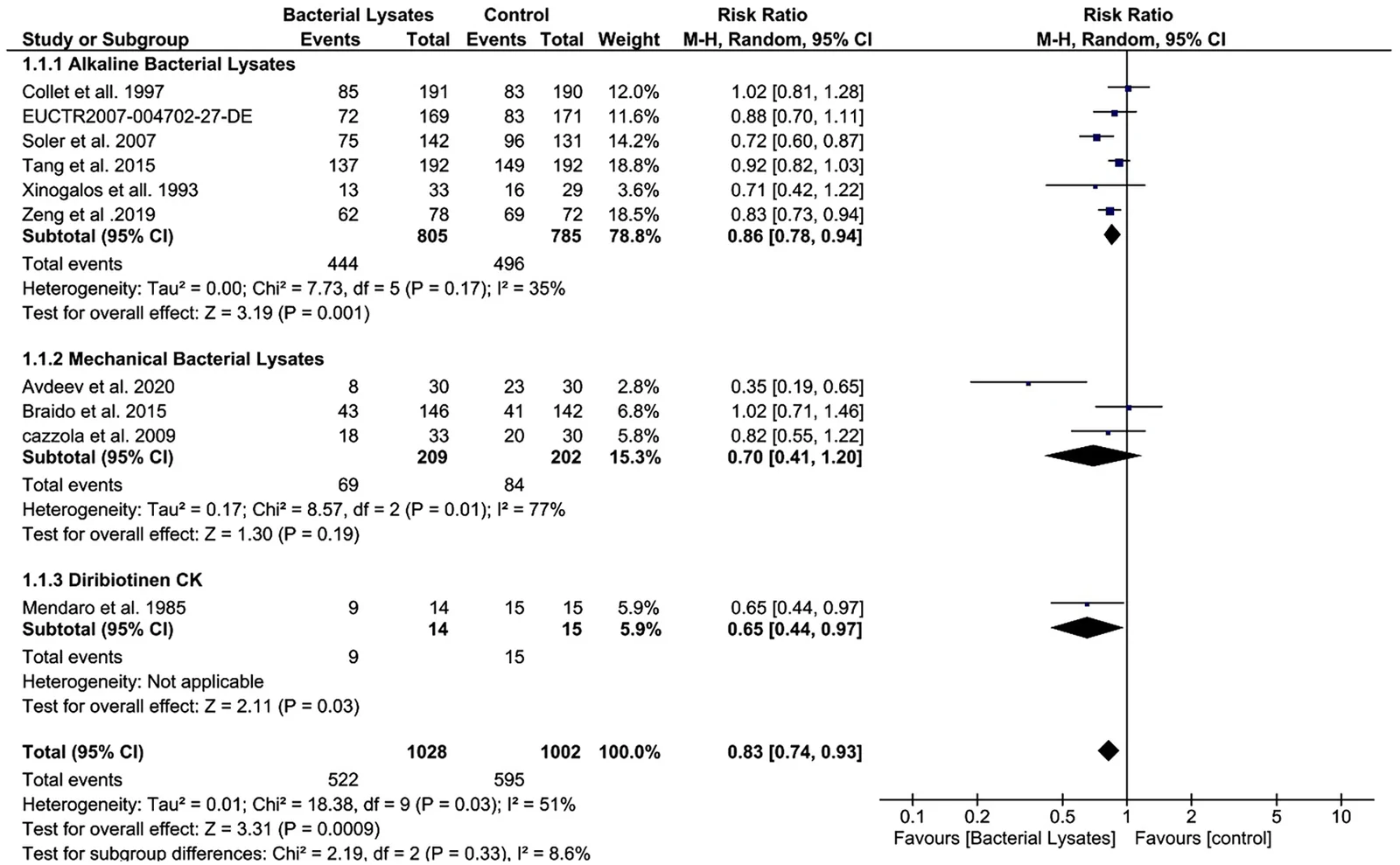
Forest plot presents the effect of using BL on exacerbation. BLs subjects vs. control subjects (RR) (9, 11, 12, 20, 23, 25, 28, 30–32).

An additional analysis evaluated the efficacy of BLs across disease severities based on FEV_1_ (% predicted). Patients with FEV_1_ ≥ 50% showed a positive benefit RR 0.88 (95% CI 0.78 to 0.99; *P* = 0.03). Heterogeneity *I*^2^ = 36%, while those with FEV_1_ < 50% demonstrated a larger effect estimate RR 0.83 (95% CI 0.74 to 0.93; *P* = 0.002). With no observe heterogeneity *I*^2^ = 0%. The overall pooled analysis showed the benefit of BLs in reducing exacerbations across disease stages RR 0.86 (95% CI 0.80 to 0.94; *P* = 0.0004), with no heterogeneity *I*^2^ = 20%. Figure 5 presents the forest plot of the efficacy of BLs across disease severities based on FEV_1_ (%predicted) (9, 11, 12, 23, 28, 30–32).

**Figure 5:**
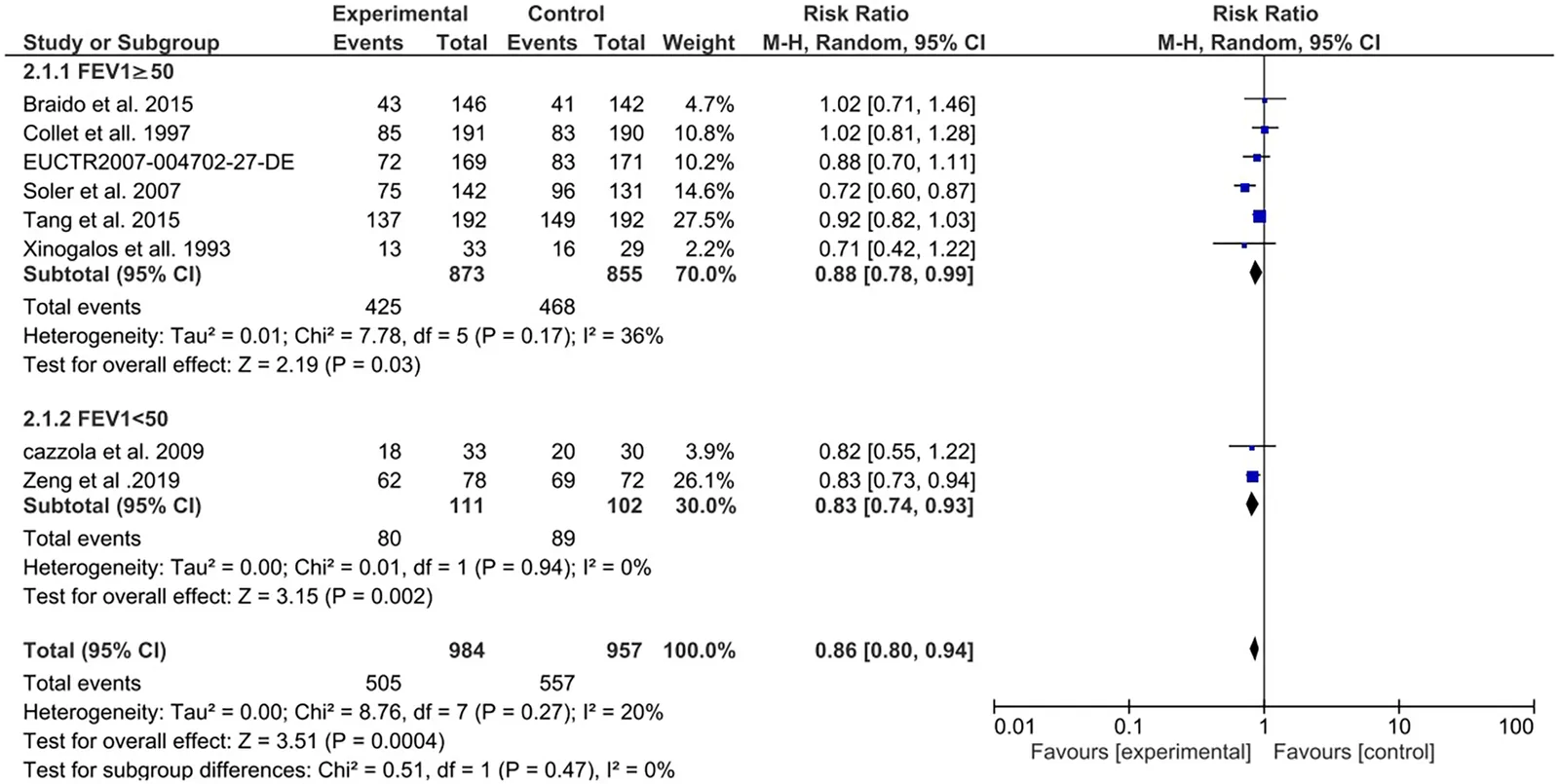
Forest plot presents the effect of using BL on exacerbation stratified by FEV_1_ (% predicted) BLs subjects vs. control subjects (RR) (9, 11, 12, 20, 23, 28, 30–32).

### 2.3 Mean number of exacerbations

Five RCTs evaluated the mean number of respiratory exacerbations. The pooled analysis showed a statistically significant reduction in the mean number of exacerbations among patients receiving bacterial lysates compared with control MD −0.19 events (95% CI −0.30 to −0.08; *P* = 0.0009). Statistical heterogeneity was low *I*^2^ = 30%. Figure 6 presents the forest plot of the mean number of exacerbations (9, 26, 28, 30, 31).

**Figure 6:**
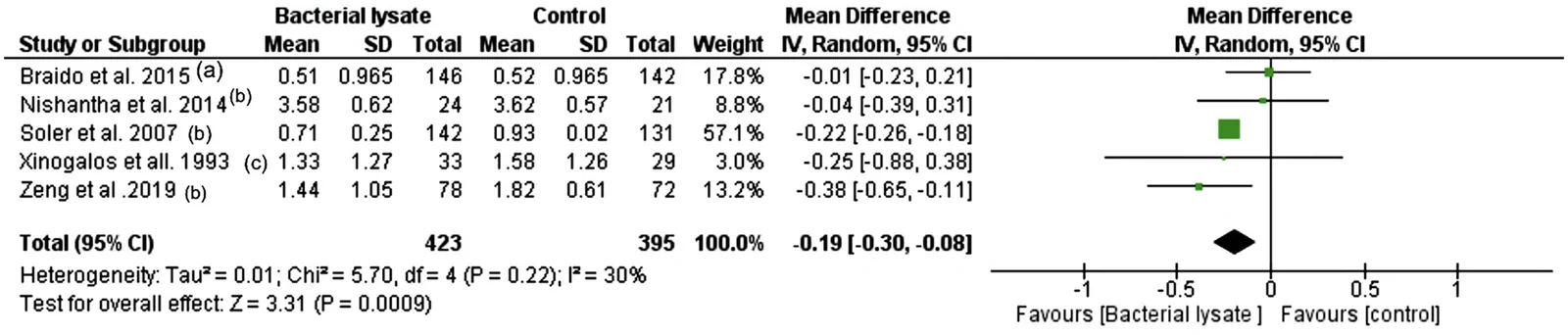
Forest plot of the mean number of exacerbations during follow-up (BLs subjects vs. control subjects; MD). Follow-up duration: **(a)** 12 months; **(b)** 6 months; **(c)** 5 months (9, 26, 28, 30, 31).

Duration of exacerbations tended to be shorter in patients treated with bacterial lysates compared with controls, although the pooled mean difference did not reach statistical significance. MD −9.26 days (95% CI −19.56 to 1.03; *P* = 0.08). Considerable heterogeneity was observed (*I*^2^ = 97%). limiting the precision of this estimate. Figure 7 presents the forest plot of the duration of exacerbations (24, 25, 27, 33).

**Figure 7:**
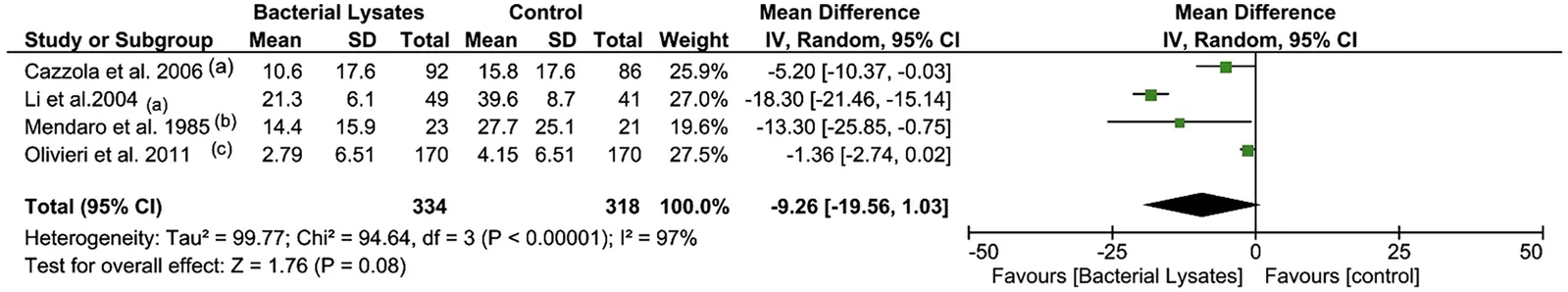
Forest plot of duration of exacerbation BLs subjects vs. control subjects (mean days). Follow-up duration: **(a)** 12 months; **(b)** 3 months; **(c)** 5 months (24, 25, 27, 33).

### 2.4 Hospitalisation

Five RCTs reporting number of hospitalisations were included. Pooled results showed alkaline BLs were not associated with reduced hospitalisations. In contrast, PMBL were associated with a 55% reduction in hospitalisation events. The overall pooled analysis for both BL types revealed a 48% decrease in hospitalisation risk RR 0.52 (95% CI 0.35 to 0.78; *P* = 0.01). With moderate heterogeneity *I*^2^ = 53%. Figure 8 presents the forest plot of hospitalisation events (11, 12, 20, 22, 33).

**Figure 8:**
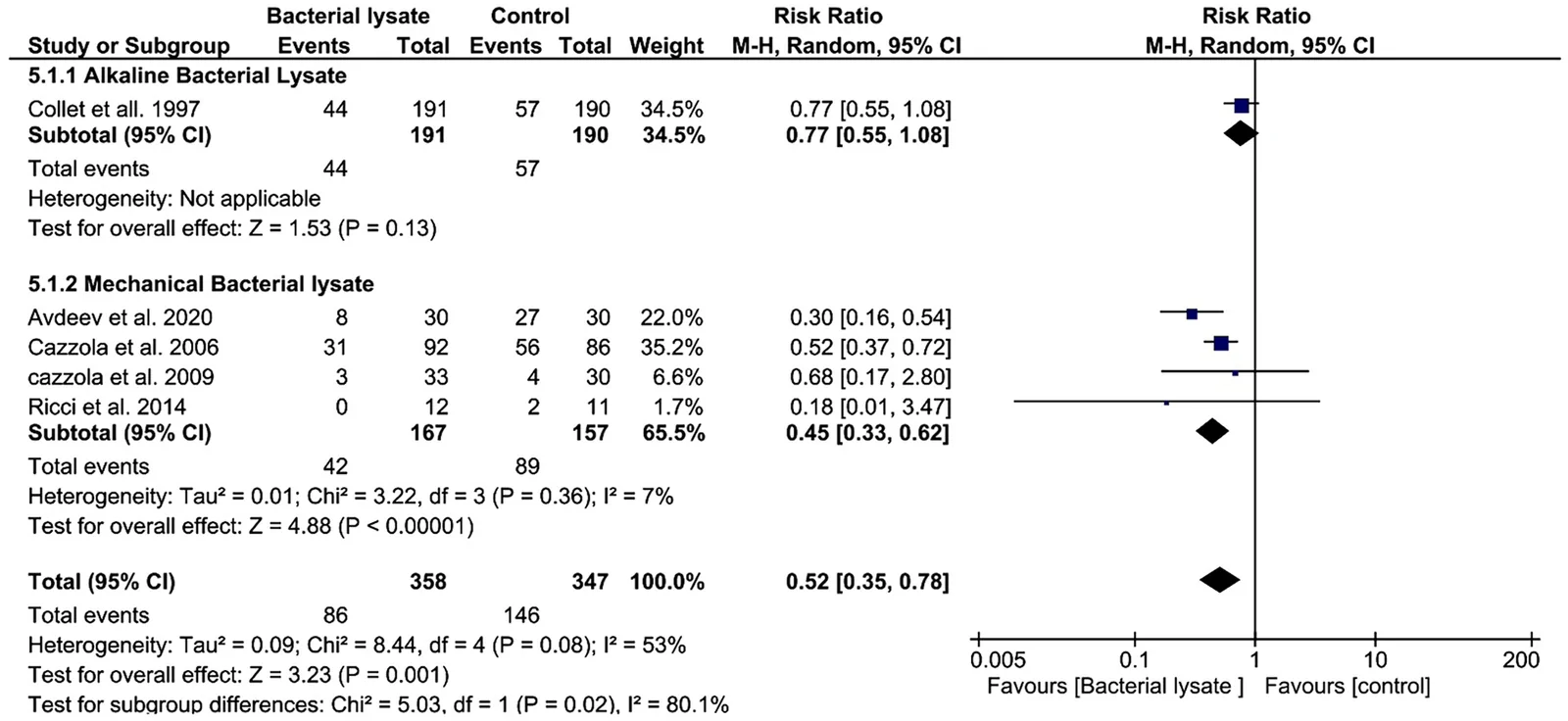
Forest plot of hospitalisations; BLs subjects vs. control subjects (RR) (11, 12, 20, 22, 33).

The pooled Hospitalisation duration tended to be shorter in patients treated with bacterial lysates compared to controls, although the overall difference did not reach statistical significance MD −3.02 days (95% CI −6.22 to 0.18; *P* = 0.06). Heterogeneity was substantial *I*^2^ = 82%, limiting the precision of this estimate. Figure 9 presents the forest plot of the hospitalisation duration (12, 33).

**Figure 9:**
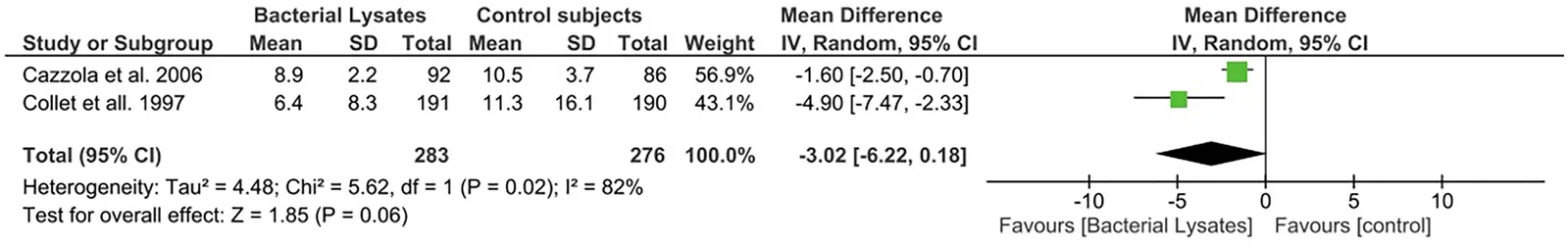
Forest plot of hospitalisation duration mean BLs subjects vs. control subjects (MD, days). Follow-up duration: **(a)** 12 months; **(b)** 3 months (12, 33).

### 2.5 Symptoms

Our analysis also assessed the effects of BLs on symptoms such as dyspnoea, sputum production, and cough. Across three studies, BLs were associated with lower dyspnoea scores compared with control SMD −0.42 (95% CI −0.75 to −0.10; *P* = 0.01). With moderate heterogeneity *I*^2^ = 54%. Figure 10 presents the forest plot of the efficacy of BLs on dyspnoea (11, 20, 24, 31).

**Figure 10:**
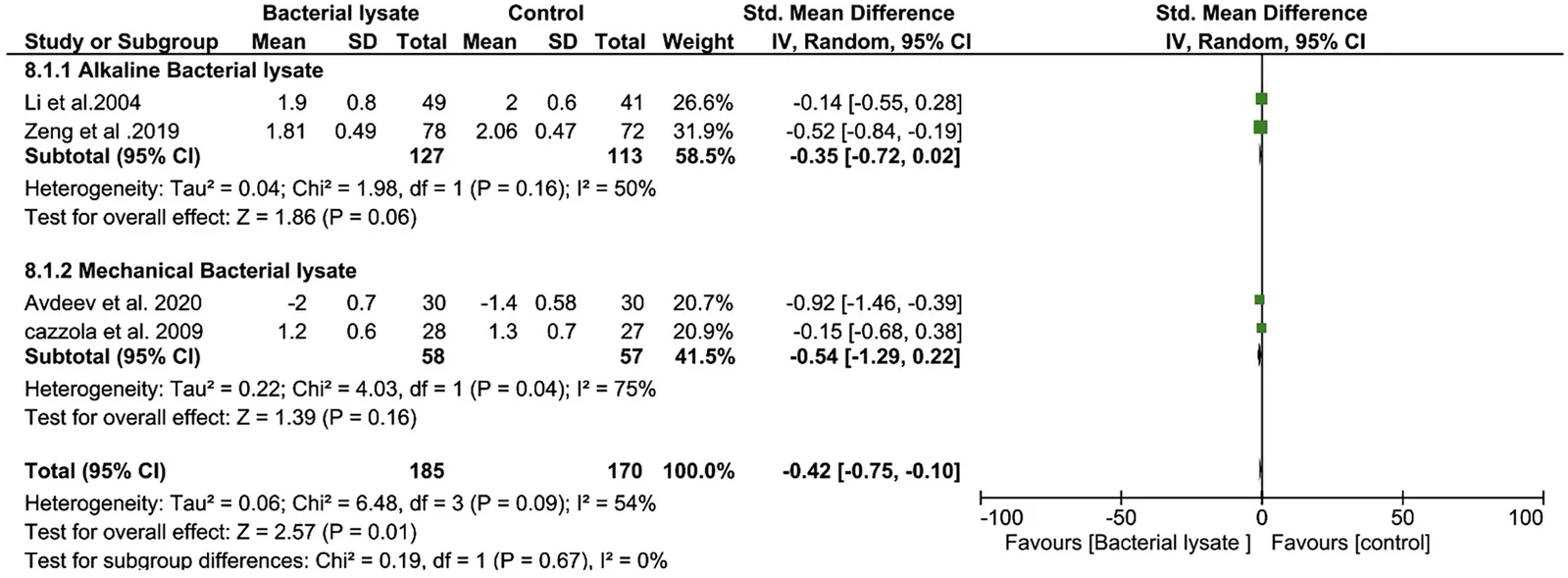
Forest plot of the efficacy of BLs on dyspnoea BLs subjects vs. control subjects (11, 20, 24, 31).

Sputum production was not significantly different between groups SMD −0.20 (95% CI −0.65 to 0.25; *P* = 0.39). With moderate heterogeneity *I*^2^ = 61%. Figure 11 presents the forest plot of the efficacy of BLs on sputum production (11, 20, 24).

**Figure 11:**
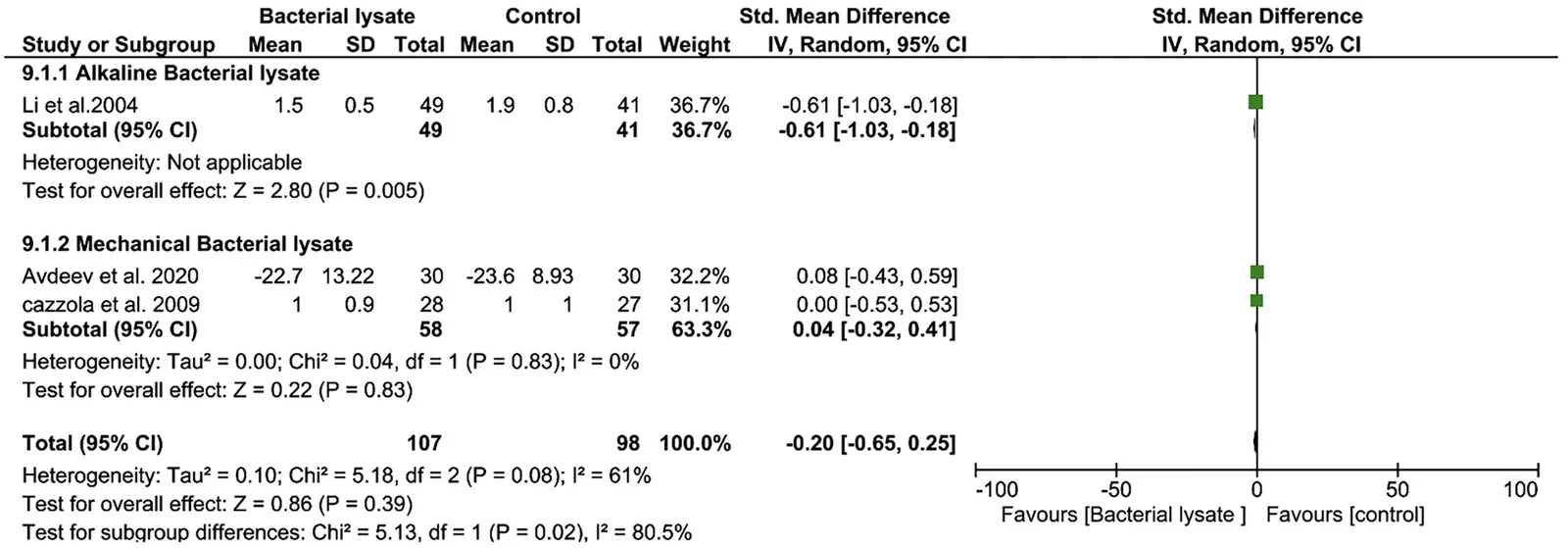
Forest plot of the efficacy of BLs on sputum production BLs subjects vs. control subjects (11, 20, 24).

Treatment with bacterial lysates was associated with a reduction in cough severity compared with control treatment SMD −0.37 (95% CI −0.63 to −0.11; *P* = 0.006). With low heterogeneity *I*^2^ = 18%. Figure 12 presents the forest plot of the efficacy of BLs on cough (11, 24, 31).

**Figure 12:**
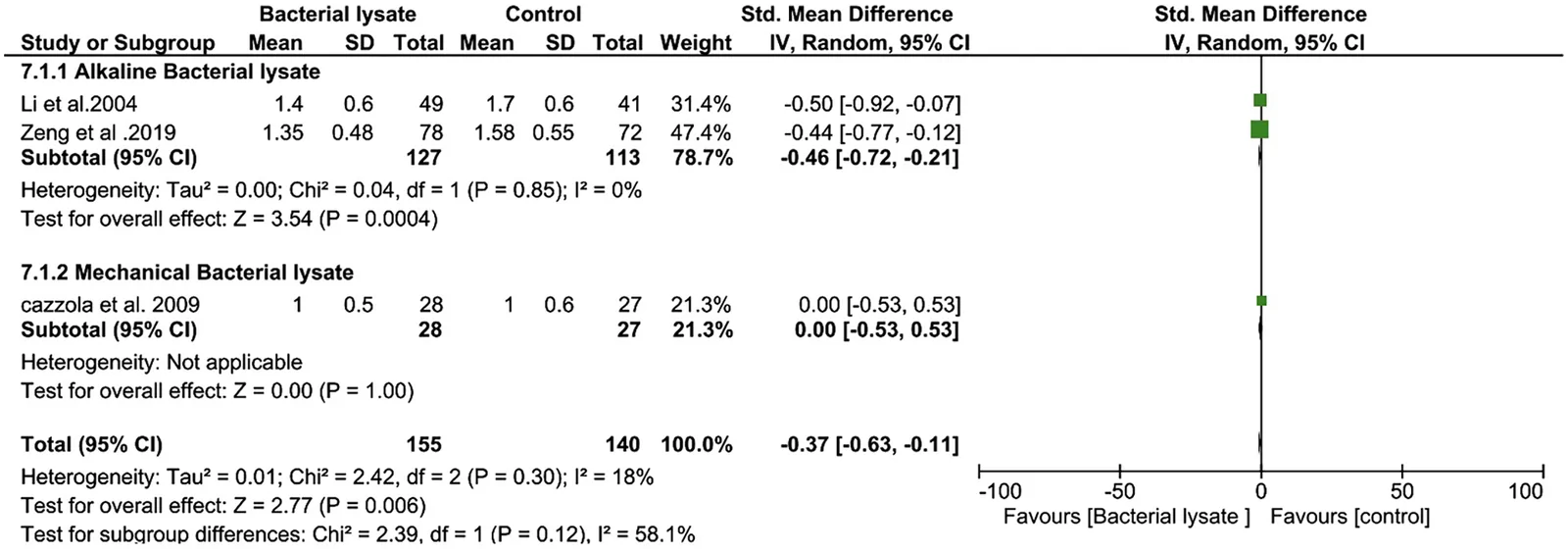
Forest plot of the efficacy of BLs on cough BLs subjects vs. control subjects (11, 24, 31).

### 2.6 Safety profile of bacterial lysates

Eight randomized controlled trials reported adverse events. No statistically significant difference in the risk of adverse events was observed between participants receiving bacterial lysates and control treatment RR 0.95 (95% CI 0.86 to 1.04; *P* = 0.27). With no heterogeneity *I*^2^ = 0%. Sensitivity analyses produced similar estimates, supporting the robustness of the overall safety finding. Figure 13 presents the forest plot of adverse events (9, 12, 23, 24, 27, 28, 31, 32).

**Figure 13:**
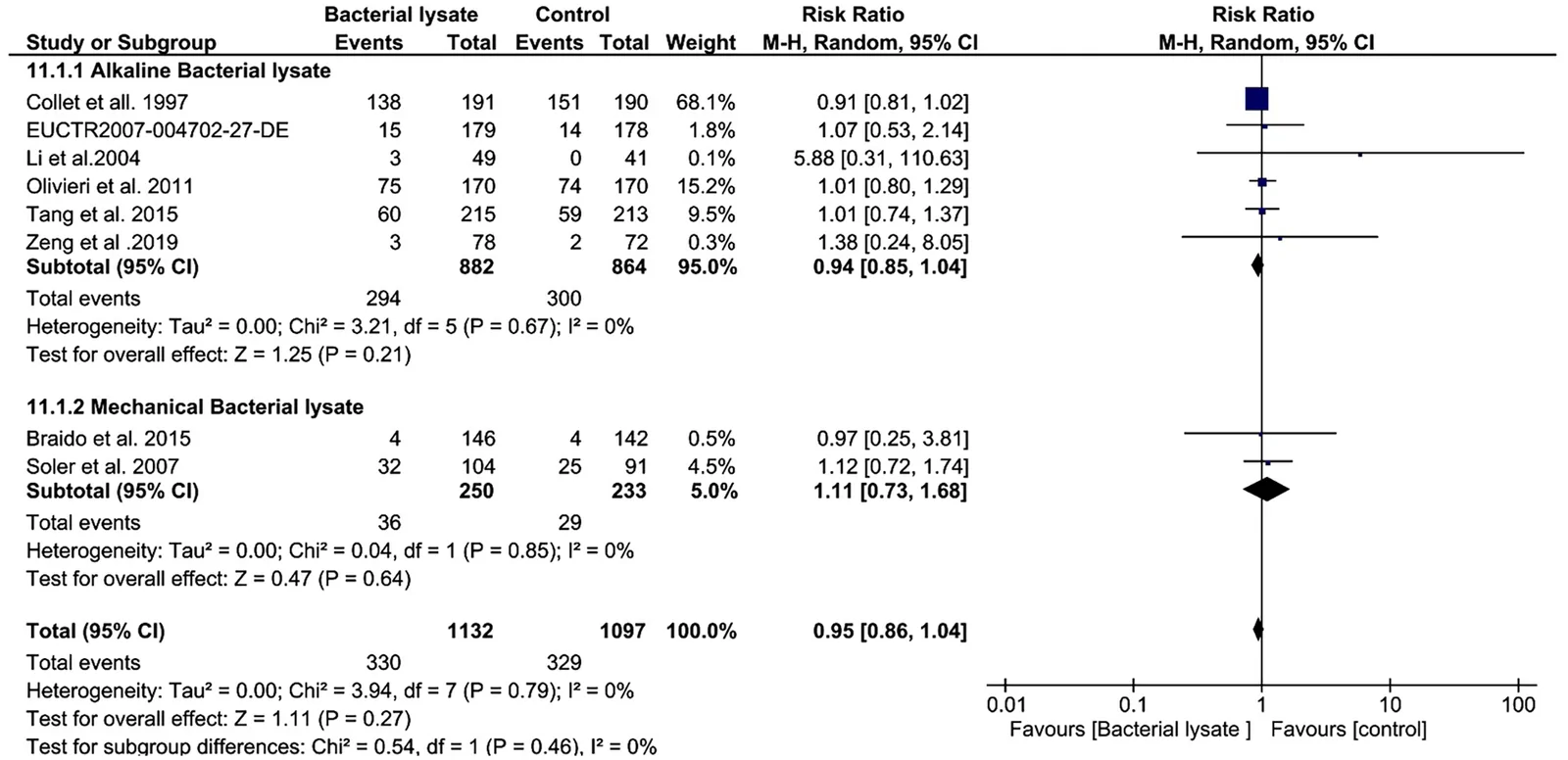
Forest plot of BLs adverse events VS. control events (safety profile) (9, 12, 23, 24, 27, 28, 31, 32).

### 2.7 Sensitivity analysis and publication bias

Sensitivity analyses using fixed and random effects models produced broadly consistent estimates for the principal outcomes. For example, the pooled estimate for exacerbations remained statistically significant under both models random-effects RR 0.83 (95% CI 0.74 to 0.93); fixed-effects RR 0.85 (95% CI 0.79 to 0.92). Similarly, the reduction in hospitalisation remained statistically significant when the fixed-effects model was applied RR 0.57 (95% CI 0.46 to 0.71). Sensitivity analyses were also conducted by excluding studies judged to have a high risk of bias. Detailed sensitivity analyses are presented in Table 1.

**Table 1:** Presents the sensitivity analyses.

A. Sensitivity analysis using different effect models (random effects and fixed effects).		
Outcome	Model (estimated value 95% CI)	
	Random effect	Fixed effect
Exacerbations (RR)	0.83 [0.74, 0.93]	0.85 [0.79, 0.92]
Hospitalisation (RR)	0.52 [0.35, 0.78]	0.57 [ 0.46, 0.71]
Mean number of exacerbations (MD)	−0.19 [−0.30, −0.08]	−0.21 [ −0.25, −0.17]
Sputum (SMD)	−0.20 [−0.65, 0.25]	−0.23 [−0.51, 0.04]
Cough (SMD)	−0.37 [−0.63, −0.11]	−0.37 [−0.61, −0.14]
Severity of dyspnoea (SMD)	−0.42 [−0.75, −0.10]	−0.42 [−0.64, −0.21]
Adverse events (RR)	0.95 [0.86, 1.04]	0.98 [0.88, 1.10]

B. Sensitivity analysis excluding studies from the primary outcomes that were assessed as having a high risk of bias.				
Outcome	Exclude Avdeev et al. (20) (Random effect)	Exclude EUCTR2007-004702-27-DE (23) (Random effect)	Exclude Menardo et al. (25) (Random effect)	Exclude Zeng et al. (31) (Random effect)
Exacerbations (RR).	0.85 [0.89, 0.93]	0.82 [0.72, 0.93]	0.84 [0.75, 0.94]	0.82 [0.71, 0.94]
*N* = 2001 participants	*N* = 1970	*N* = 1690	*N* = 2001	*N* = 1880
*K* = 10 trials	*K* = 9	*K* = 9	*K* = 9	*K* = 9
Hospitalisation. (RR)	0.62 [0.48, 0.81]	N/A	N/A	N/A
*N* = 705participants	*N* = 645			
*K* = 5trials	*K* = 4			
Mean number of exacerbations (MD).	N/A	N/A	N/A	−0.16 [−0.28, −0.04]
*N* = 818participants				*N* = 668
*K* = 5trials				*K* = 4
Symptoms score sputum (SMD)	−0.3 [−0.92, 0.27]	N/A	N/A	N/A
*N* = 205participants	*N* = 145			
*K* = 3trials	*K* = 2			
Symptoms score Cough (SMD)	N/A	N/A	N/A	−0.27 [−0.76, 0.21]
*N* = 295 participants				*N* = 145
*K* = 3 trials				*K* = 2
Severity of dyspnoea (SMD)	−0.32 [−0.58, −0.05]	N/A	N/A	−0.39 [−0.88, 0.10]
*N* = 355participants	*N* = 295			*N* = 205
*K* = 4 trials	*K* = 3			*K* = 3
Adverse events (RR)	N/A	0.95 [0.86, 1.04]	N/A	0.95 [0.86, 1.04]
*N* = 2229 participants		*N* = 1872		*N* = 2079
*K* = 8 trials		*K* = 7		*K* = 7

Publication bias was assessed using a funnel plot in Figure 14. Although some visual asymmetry was observed, neither Egger's nor Begg–Mazumdar's test showed significant asymmetry (*P* > 0.15). The trim-and-fill method identified only one potentially missing study, with a minimal change in the pooled effect estimate to (RR = 0.85).

**Figure 14:**
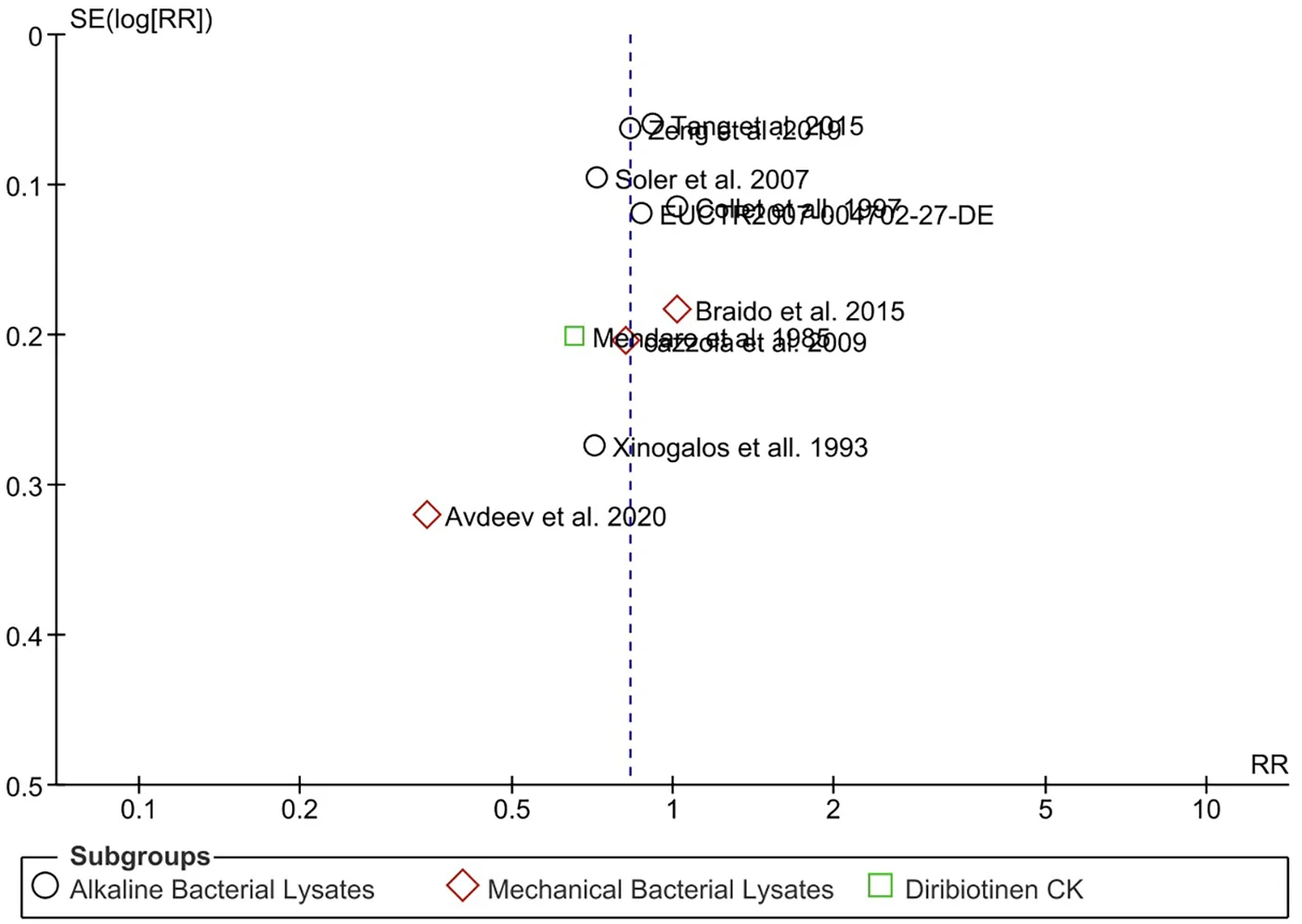
Funnel plot of the effect of using BL on exacerbation.

## 3 Discussion

The principal finding of this systematic review and meta-analysis elucidates the potential advantages of BLs in the management of COPD, including reduction of exacerbations, hospitalisations, symptoms score, and adverse events, while also recognizing their limitations.

The potential protective effect of BLs on COPD exacerbations is thought to rely on their ability to mimic natural microbial exposure, stimulating both innate and adaptive immune responses. These preparations, obtained through mechanical, chemical or other lytic techniques interact with the mucosal immune system through mechanisms involving the gut–lung immunological axis (34). Previous experimental and clinical literature suggests that these preparations can influence dendritic cells, macrophages, and epithelial cells via TLR-2 and TLR-4 (34, 35). BLs act as pathogen-associated molecular patterns on innate immunity and as antigens on adaptive immunity, promoting cytokine (IL-1, IL-6, TNF-α) and chemokine (IL-8, CCL2, CCL20) release, stimulation of NK and CD8⁺ cytotoxic cells and mucosal IgA production. These antibodies may facilitate the clearance of the pathogens by innate effector cells, while also promoting a broader Th1-type immune response. Overall, these effects support the concept of “trained immunity”, suggesting that BLs maintain the immune system highly prepared possibly resulting in a lower frequency of recurrent respiratory infections reported in treated populations (9, 21, 34–37).

In assessing the effect of BLs on exacerbation risk, BL treatment showed a decrease in exacerbations by 17% compared with placebo RR 0.83 (95% CI: 0.74 to 0.93; *P* = 0.0009). With moderate heterogeneity *I*^2^ = 51%. This was driven by alkaline BLs which showed a statistically favorable outcome RR 0.86 (95% CI 0.78 to 0.94; *P* = 0.001). *I*^2^ = 35%, and Diribiotine CK RR 0.65 (95% CI: 0.44 to 0.97; *P* = 0.03), compared to PMBL which did not show a significant reduction RR 0.70 (95% CI 0.41 to 1.20; *P* = 0.19). This finding is likely influenced by the small sample size and substantial heterogeneity (*I*^2^ = 77%) among available studies in PMBL group, which limits statistical precision and reduces confidence in the pooled estimate. While methodological differences in lysate preparation may also contribute to the observed discrepancy, the current evidence base for mechanical lysates remains insufficient to draw firm conclusions (34, 38). However, the present finding is broadly consistent with previous evidence suggesting that BLs may reduce recurrent respiratory exacerbations. Huang et al. (45) similarly reported a reduction in the mean number of exacerbations among patients receiving BLs, with a greater effect observed with alkaline formulations. The agreement between these findings supports the possibility that BLs may provide an adjunctive preventive effect rather than serving primarily as treatment for established acute exacerbations.

An additional analysis stratified by baseline FEV_1_ suggested better protective effect of BLs in more severe patients. Participants with FEV_1_ ≥ 50% tended to show a positive benefit RR 0.88 (95% CI: 0.78 to 0.99; *P* = 0.03), but those with FEV_1_ < 50% experienced a larger effect RR 0.83 (95% CI: 0.74 to 0.93; *P* = 0.002). With overall no heterogeneity (*I*^2^ = 20%). This finding was based on a limited number of studies and should be regarded as exploratory rather than as evidence of differential treatment efficacy according to disease severity.

Our analysis showed a significant reduction in the mean number of exacerbations compared with control over study period, with a pooled MD of −0.19 Events (95% CI: −0.30 to −0.08; *P* = 0.0009). Although the overall effect size was modest, the low heterogeneity (Chi^2^ = 5.70, df = 4, *P* = 0.22; *I*^2^ = 30%) suggests that the benefit is reproducible. Since each episode affects symptom burden, antibiotic exposure, and healthcare utilization, even a slight decrease in exacerbations may be clinically relevant. These findings are consistent with the findings of Huang et al. (45) who also reported that BLs were associated with fewer exacerbations MD −0.42 (95% CI: −0.75 to −0.08). Particularly noting a greater effect with alkaline lysates.

Despite the lack of statistical significance, the reduction in exacerbation duration MD −9.26 days (95% CI: −19.56 to 1.03; *P* = 0.08) was associated with significant heterogeneity (*P* < 0.00001; *I*^2^ = 97%). To explore this further, a sensitivity analysis was conducted. After excluding Li et al. (24), MD −4.05 days (95% CI: −8.83 to 0.73; *P* = 0.10) heterogeneity decreased (Tau^2^ = 10.44; Chi^2^ = 5.27, df = 2, *P* = 0.07; *I*^2^ = 62%).

These results suggest that BLs may be considered for further evaluation as an adjuvant preventive intervention by demonstrating that they effectively lower exacerbations, especially in individuals with more severe disease (FEV_1_ < 50%), rather than shortening the duration of exacerbations.

The pooled analysis of five randomized controlled trials (*N* = 705 participants) showed that BLs reduced hospitalisation compared to control group. Alkaline lysates showed no significant advantage RR 0.77 (95% CI: 0.55 to 1.08; *P* = 0.13). Although their effect on the risk of exacerbation was significant, likely reflecting small sample sizes (*K* = 1) among available trials which limit our statistical precision. In contrast, PMBL reduced hospitalisation risk RR 0.45 (95% CI: 0.33 to 0.62; *P* = <0.00001), with a significant difference between subgroups (*P* = 0.02). However, the observed difference between bacterial lysate formulations is hypothesis-generating and may reflect differences in formulation, treatment regimen, patient characteristics, or other study-level factors (10, 11, 33, 39).

The results of hospitalisation duration analysis were less consistent. The high heterogeneity (*I*^2^ = 82%) limited pooled interpretation. The included trials (12, 33) revealed non-significant reduction in the mean-days respiratory-related hospital stays among lysate-treated patients (12, 33). While Collet et al. (12) observed a reduction with OM-85 MD −4.90 (95% CI: −7.47 to −2.33), Cazzola et al. (33) showed a mean hospital stay with Ismigen SMD −1.60 (95% CI: −2.50 to −0.70) (11, 12, 33). Pooled results showed MD −3.02 (95% CI: −6.22 to 0.18; *P* = 0.06). Variations in findings between studies may be due to follow-up duration, disease severity, different sample size, Immune response which may be less sensitive to treatment effect than hospitalisation events.

Overall, BLs were associated with improvement in respiratory symptom scores when compared with control, including reduction in cough, dyspnoea, but not in sputum production. There was a noticeable difference between the lysate preparation techniques when comparing between subgroups. Alkaline bacterial lysates consistently produced positive improvements, particularly for cough and sputum symptoms. The subgroup included studies with general consistency and low heterogeneity. In contrast, PMBL did not significantly differ from control.

The safety analysis of bacterial lysates suggests that their safety profile is comparable to that of controls with no significant increase in adverse events. This highlights their tolerability compared with other commonly used therapies and their suitability for long-term prophylactic use.

Previous studies of BLs and recurrent respiratory infections have reported reductions in infection frequency and, in some populations, reductions in antibiotic use and infection duration (40–42). These findings provide additional support for the concept that BLs may influence susceptibility to recurrent respiratory infections. However, much of this evidence comes from populations outside COPD, Schaad et al. reported that preventive efficacy was proportionally higher in children with a recurrent respiratory tract infection. Highlighting how immunomodulators such as BLs can offer more pronounced protection in highly susceptible individuals. Similarly, Gutierrez-Tarango noted a decrease in infection rates and also a shorter duration of infection (35.23 ± 17.64 vs. 60.75 ± 25.44 days), fewer prescriptions for antibiotics (2.46 ± 2.08 vs. 4.46 ± 2.08), suggesting broader clinical benefits than just incidence reduction (40–42). Antibiotics are routinely prescribed to treat infections in Patients with COPD; their frequent use has contributed to the rising antibiotic resistance, which reduces the effectiveness of existing treatments. Additionally, antibiotics disrupt immune responses by reducing exposure to antigens and disrupting the normal bacterial flora that help regulate inflammation, making their benefits short-lived. BLs have been shown to stimulate both innate and adaptive immune system responses and may aid in strengthening defense against recurrent infections. Although current findings are encouraging, further research directly comparing BLs with antibiotics is needed to clarify their potential effects in COPD management (40, 43, 44).

The present investigation has several limitations. First, while the meta-analysis only included RCTs to minimize bias, many studies involved short to medium follow-up periods. When exacerbation outcomes were assessed over less than 6 months, any observed treatment effect may have been limited. The analysis was based on a relatively small number of studies (*K* = 15). Many of which were published several years ago, which may affect the applicability of findings to current clinical practice. Heterogeneity was detected in the overall analysis but was reduced through subgroup analyses, stratified by BLs type (PMBL vs. alkaline vs. Diribiotine CK) and by lung function (FEV_1_ ≥ 50% vs. <50%). The reduced heterogeneity within these subgroups suggests that the variability in the overall results is likely due to differences between the subgroups rather than inconsistencies within them. To evaluate the reliability of the findings, we performed a sensitivity analysis and subsequently graded the certainty of the evidence (see Summary of findings in Table 2). The variability in how outcomes were reported made it challenging to extract data. Although clear inclusion criteria were applied to ensure consistency, the study protocols varied, and standardised outcome measures were not used. For example, Collet et al. reported a dyspnoea score, but this outcome was excluded from our analysis because a different measurement tool (Oxygen Cost Diagram, McGavin) was used (12).

**Table 2:** Grade summary of findings for the main outcomes.

Author(s): Question: [bacterial lysates] compared to [placebo] for [COPD] Setting: Bibliography:												
No. of studies	Study design	Risk of bias	Inconsistency	Indirectness	Imprecision	Other considerations	No. of patients [bacterial lysates]	No. of patients [placebo]	Relative (95% CI)	Absolute (95% CI)	Certainty	Importance
Rate of exacerbation [follow-up: range 3–12 months; assessed with: number of patients with ≥1 exacerbation—RR (95% CI)]												
10	Randomised trials	Not serious	Serious^a^	Not serious	Not serious	None	522/1028 (50.8%)	595/1002 (59.4%)	RR 0.83 (0.74 to 0.93)	101 fewer per 1,000 (from 154 fewer to 42 fewer)	⊕⊕⊕○ Moderate^a^	CRITICAL
Hospitalization (follow-up: range 3–12 months: assessed with: patients hospitalised ≥1 time [events/total; RR (95% CI)]												
5	Randomised trials	Not serious	Serious^b^	Not serious	Not serious	None	86/358 (24.0%)	146/347 (42.1%)	RR 0.52 (0.35 to 0.78)	202 fewer per 1,000 (from 273 fewer to 93 fewer)	⊕⊕⊕○ Moderate^b^	CRITICAL
Exacerbation mean (follow-up: range 5–12 months; assessed with: mean of exacerbation per patient)												
5	Randomised trials	Not serious	Serious	Not serious	Not serious	None	423	395	–	MD 0.19 SD lower (0.3 lower to 0.08 lower)	⊕⊕○○ Low	CRITICAL
Symptoms score (dyspnea) (follow-up: range 3–12 months)												
4	Randomised trials	Serious	Serious	Not serious	Not serious	None	185	170	–	MD 0.42 Symptoms score lower (0.75 lower to 0.1 lower)	⊕⊕○○ Low	CRITICAL
Symptoms score (sputum production) (follow-up: range 3–12 months)												
3	Randomised trials	Serious	Serious	Not serious	Not serious	None	107	98	–	MD 0.2 Sputum production lower (0.65 lower to 0.25 higher)	⊕⊕○○ Low	CRITICAL
Symptom score (cough)												
3	Randomised trials	Serious	Not serious	Not serious	Not serious	None	155	140	–	MD 0.37 Cough lower (0.63 lower to 0.11 lower)	⊕⊕⊕○ Moderate	CRITICAL
Safety and side effect												
8	Randomised trials	Not serious	Not serious	Not serious	Not serious	None	330/1,132 (29.2%)	329/1,097 (30.0%)	RR 0.95 (0.86–1.04)	15 fewer per 1,000 (from 42 fewer to 12 more)	⊕⊕⊕⊕ High	CRITICAL

CI, confidence interval; MD, mean difference; RR, risk ratio.

a Exacerbation rate: slight visual inconsistency and analysis shows heterogeneity.

b Hospitalization rate + dyspnea: analysis shows heterogeneity.

Future research should focus on direct comparisons of different BLs formulations, Large-scale, rigorously designed RCTs are needed to confirm the benefits of BLs across COPD stages.

## 4 Conclusion

The systematic review and meta-analysis examined the therapeutic benefit and risk of BLs in treating Patients with COPD. BLs, particularly alkaline formulation, showed promise in reducing exacerbation. Additionally, BLs may reduce hospitalisation and have potential benefit in manage symptoms like dyspnoea and cough, but certainty is limited, and results should be interpreted cautiously. BL showed similar safety profile to the control drug.

Bacterial lysates may be associated with reduced COPD exacerbations and hospitalisations, indicating a potential preventive benefit for patients at risk of recurrent disease worsening.

## Data Availability

The original contributions presented in the study are included in the article/Supplementary Material, further inquiries can be directed to the corresponding author.

## References

[1] (GOLD) GIfCOLD. Global strategy for the diagnosis, management, and prevention of chronic obstructive pulmonary disease: 2025 report. (2024).

[2] Christenson SA Smith BM Bafadhel M Putcha N. Chronic obstructive pulmonary disease. Lancet. (2022) 399(10342):2227–42. 10.1016/S0140-6736(22)00470-6 35533707

[3] Celli Bartolomé R Wedzicha Jadwiga A. Update on clinical aspects of chronic obstructive pulmonary disease. N Engl J Med. (2019) 381(13):1257–66. 10.1056/NEJMra1900500 31553837

[4] Carlin BW. Exacerbations of COPD. Respir Care. (2023) 68(7):961–72. 10.4187/respcare.10782 37353338 PMC10289624

[5] Chou C-H Chen Y-F Peng H-C Chen C-Y Cheng B-W. Environmental pollutants increase the risks of acute exacerbation in patients with chronic airway disease. Front Public Health. (2023) 11:2023. 10.3389/fpubh.2023.1215224 PMC10643209 38026400

[6] Choi J Shim JJ Lee MG Rhee CK Joo H Lee JH. Association between air pollution and viral infection in severe acute exacerbation of chronic obstructive pulmonary disease. J Korean Med Sci. (2023) 38(9):e68. 10.3346/jkms.2023.38.e68 36880109 PMC9988434

[7] Wedzicha JA Calverley PMA Albert RK Anzueto A Criner GJ Hurst JR. Prevention of COPD exacerbations: a European respiratory society/American thoracic society guideline. Eur Respir J. (2017) 50(3):1602265. 10.1183/13993003.02265-2016 28889106

[8] Ge Y Tang G Fu Y Deng P Yao R. The impact of environmental factors on respiratory tract microbiome and respiratory system diseases. Eur J Med Res. (2025) 30(1):236. 10.1186/s40001-025-02517-3 40186246 PMC11970004

[9] Braido F Melioli G Cazzola M Fabbri L Blasi F Moretta L. Sub-lingual administration of a polyvalent mechanical bacterial lysate (PMBL) in patients with moderate, severe, or very severe chronic obstructive pulmonary disease (COPD) according to the GOLD spirometric classification: a multicentre, double-blind, randomised, controlled, phase IV study (AIACE study: advanced immunological approach in COPD exacerbation). Pulm Pharmacol Ther. (2015) 33:75–80. 10.1016/j.pupt.2015.03.006 25951945

[10] Cazzola M Capuano A Rogliani P Matera MG. Bacterial lysates as a potentially effective approach in preventing acute exacerbation of COPD. Curr Opin Pharmacol. (2012) 12(3):300–8. 10.1016/j.coph.2012.01.019 22365728

[11] Cazzola M Noschese P Perna FD. Value of adding a polyvalent mechanical bacterial lysate to therapy of COPD patients under regular treatment with salmeterol/fluticasone. Ther Adv Respir Dis. (2009) 3(2):59–63. 10.1177/1753465809104677 19443519

[12] Collet JP Shapiro P Ernst P Renzi T Ducruet T Robinson A. Effects of an immunostimulating agent on acute exacerbations and hospitalizations in patients with chronic obstructive pulmonary disease. The PARI-IS study steering committee and research group. Prevention of acute respiratory infection by an immunostimulant. Am J Respir Crit Care Med. (1997) 156(6):1719–24. 10.1164/ajrccm.156.6.9612096 9412546

[13] Page MJ McKenzie JE Bossuyt PM Boutron I Hoffmann TC Mulrow CD. The PRISMA 2020 statement: an updated guideline for reporting systematic reviews. Br Med J. (2021) 372:n71. 10.1136/bmj.n71 PMC8005924 33782057

[14] Ouzzani M Hammady H Fedorowicz Z Elmagarmid A. Rayyan—a web and mobile app for systematic reviews. Syst Rev. (2016) 5(1):210. 10.1186/s13643-016-0384-4 27919275 PMC5139140

[15] Chandler J Cumpston M Li T Page MJ Welch V. Cochrane Handbook for Systematic Reviews of Interventions. Hoboken: Wiley (2019), 4(1002):14651858.

[16] Higgins JP Sterne JA Savovic J Page MJ Hróbjartsson A Boutron I. A revised tool for assessing risk of bias in randomized trials. Cochrane Database Syst Rev. (2016) 10(Suppl 1):29–31.

[17] Sterne JAC Savović J Page MJ Elbers RG Blencowe NS Boutron I. Rob 2: a revised tool for assessing risk of bias in randomised trials. Br Med J. (2019) 366:l4898. 10.1136/bmj.l4898 31462531

[18] The Cochrane Collaboration. Review manager (RevMan). The Cochrane Collaboration (2020).

[19] Choi GJ Kang H. Heterogeneity in meta-analyses: an unavoidable challenge worth exploring. Korean J Anesthesiol. (2025) 78:301–314. 10.4097/kja.25001 39956636 PMC12326561

[20] Avdeev SN Nuralieva GS Gainitdinova VV Baimakanova GE So AK Merzhoeva ZM. Clinical efficacy of mechanical bacterial lysate in the prevention of infectious exacerbations of chronic obstructive pulmonary disease. Ter Arkh. (2020) 92(4):57–63. 10.26442/00403660.2020.04.000590 32598699

[21] Braido F Tarantini F Ghiglione V Melioli G Canonica GW. Bacterial lysate in the prevention of acute exacerbation of COPD and in respiratory recurrent infections. Int J Chron Obstruct Pulmon Dis. (2007) 2(3):335–45 PMID: 18229572; PMCID: PMC2695191. 18229572 PMC2695191

[22] Ricci R Palmero C Bazurro G Riccio AM Garelli V Di Marco E. The administration of a polyvalent mechanical bacterial lysate in elderly patients with COPD results in serological signs of an efficient immune response associated with a reduced number of acute episodes. Pulm Pharmacol Ther. (2014) 27(1):109–13. 10.1016/j.pupt.2013.05.006 23792312

[23] (EudraCT) UUCTR. Sub-lingual administration of a polyvalent mechanical bacterial lysate (PMBL) in patients with moderate or severe and very severe chronic obstructive pulmonary disease (COPD) according to GOLD classification: a multicenter, international, double blind, randomized, controlled, phase IV study (2007). 10.1016/j.pupt.2015.03.006 25951945

[24] Li J Zheng JP Yuan JP Zeng GQ Zhong NS Lin CY. Protective effect of a bacterial extract against acute exacerbation in patients with chronic bronchitis accompanied by chronic obstructive pulmonary disease. Chin Med J. (2004) 117(6):828–34 PMID: 15198881. 15198881

[25] Menardo J-L Dussol J-P Betbeder A. Immunotherapy of respiratory infection by oral immunomodulation in chronic obstructive bronchopneumopathy. Med Hyg. (1985) 43(1623):2722–6.

[26] Nishantha KM Gunaratne HC Yasaratne D Medegedara RM. Polyvalent mechanical bacterial lysate in advanced COPD; a randomized controlled trial. Respirology. (2014) 19(Suppl 3):121. 10.1111/resp.12416

[27] Olivieri D. Reducing exacerbations in COPD with OM-85: a multicentre, double-blind, placebo-controlled trial. Eur Respir J. (2011) 38(Suppl 55):202. 10.1183/13993003/erj.38.Suppl_55.202

[28] Soler M Mütterlein R Cozma G. Double-blind study of OM-85 in patients with chronic bronchitis or mild chronic obstructive pulmonary disease. Respiration. (2007) 74(1):26–32. 10.1159/000093933 16772707

[29] Tang H Fang Z Xiu Q. Efficacy and safety of bacterial lysates in patients with chronic obstructive pulmonary disease and exacerbation. European Respiratory Society (2011).

[30] Xinogalos S Duratsos D Varonos D. Clinical effectiveness of broncho-vaxom (BV) in patients with chronic obstructive pulmonary disease. Int J Immunother. (1993) 9(2):135–42.

[31] Zeng D Huang J Wang B Chen G. Efficacy of Broncho-Vaxom on chronic obstructive pulmonary disease in elderly patients. Chin J Geriatr. (2019) 38(7):717–21. 10.3760/cma.j.issn.0254-9026.2019.07.002 [in Chinese]

[32] Tang H Fang Z Saborío GP Xiu Q. Efficacy and safety of OM-85 in patients with chronic bronchitis and/or chronic obstructive pulmonary disease. Lung. (2015) 193(4):513–9. 10.1007/s00408-015-9737-3 26044556

[33] Cazzola M. A new bacterial lysate protects by reducing infectious exacerbations in moderate to very severe COPD: a double-blind, randomized, placebo-controlled trial. Trends Med. (2006) 6(3):191–199. Available online at: https://www.oatext.com/pdf/TiM-6-264.pdf

[34] Di Gioacchino M Santilli F Pession A. Is there a role for immunostimulant bacterial lysates in the management of respiratory tract infection? Biomolecules. (2024) 14(10):1249. 10.3390/biom14101249 39456182 PMC11505618

[35] Parola C Salogni L Vaira X Scutera S Somma P Salvi V. Selective activation of human dendritic cells by OM-85 through a NF-kB and MAPK dependent pathway. PLoS One. (2013) 8(12):e82867. 10.1371/journal.pone.0082867 24386121 PMC3875422

[36] Bienenstock J McDermott M Befus D O'Neill M. A common mucosal immunologic system involving the bronchus, breast and bowel. Adv Exp Med Biol. (1978) 107:53–9. 10.1007/978-1-4684-3369-2_7 742502

[37] Esposito S Soto-Martinez ME Feleszko W Jones MH Shen KL Schaad UB. Nonspecific immunomodulators for recurrent respiratory tract infections, wheezing and asthma in children: a systematic review of mechanistic and clinical evidence. Curr Opin Allergy Clin Immunol. (2018) 18(3):198–209. 10.1097/ACI.0000000000000433 29561355 PMC6037280

[38] Suárez N Ferrara F Rial A Dee V Chabalgoity JA. Bacterial lysates as immunotherapies for respiratory infections: methods of preparation. Front Bioeng Biotechnol. (2020) 8:2020. 10.3389/fbioe.2020.00545 PMC7289947 32582669

[39] Cazzola M Anapurapu S Page CP. Polyvalent mechanical bacterial lysate for the prevention of recurrent respiratory infections: a meta-analysis. Pulm Pharmacol Ther. (2012) 25(1):62–8. 10.1016/j.pupt.2011.11.002 22155205

[40] Gutiérrez-Tarango MD Berber A. Safety and efficacy of two courses of OM-85 BV in the prevention of respiratory tract infections in children during 12 months. Chest. (2001) 119(6):1742–8. 10.1378/chest.119.6.1742 11399700

[41] Jurkiewicz D Zielnik-Jurkiewicz B. Bacterial lysates in the prevention of respiratory tract infections. Otolaryngol Pol. (2018) 72(5):1–8. 10.5604/01.3001.0012.7216 30460909

[42] Schaad UB. OM-85 BV, an immunostimulant in pediatric recurrent respiratory tract infections: a systematic review. World J Pediatr. (2010) 6(1):5–12. 10.1007/s12519-010-0001-x 20143206

[43] Qi Y Zhu Z Liu X Yang J Zhang W Huang J. The effect of oral bacterial lysates on the respiratory microbiome in patients with chronic obstructive pulmonary disease exacerbations—a pilot study. Med Microecol. (2022) 14:100067. 10.1016/j.medmic.2022.100067

[44] Troiano G Messina G Nante N. Bacterial lysates (OM-85 BV): a cost-effective proposal in order to contrast antibiotic resistance. J Prev Med Hyg. (2021) 62(2):E564–73. 10.15167/2421-4248/jpmh2021.62.2.1734 34604601 PMC8451348

[45] Huang Y Pei Y Qian Y Yao Z Chen C Du J. A meta-analysis on the efficacy and safety of bacterial lysates in chronic obstructive pulmonary disease. Front Med. (2022) 9:877124. 10.3389/fmed.2022.877124 PMC9218542 35755034

