# Supplementary material for: Evaluating the therapeutic benefit of bacterial lysates in chronic obstructive pulmonary disease: a systematic review and meta-analysis
Source: Front Med (Lausanne). 2026 Oct 21;13:1928291. doi: 10.3389/fmed.2026.1928291 (PMC13731251; doi:10.3389/fmed.2026.1928291)
Supplement: Supplementary file 1 [file Table1.docx]

| Study ID (Author/Year) | Total number of patients included  (Placebo / treatment ) | Major inclusion criteria | | Route of administration | Bacterial subgroup | Treatment schedule | Type of intervention |
| --- | --- | --- | --- | --- | --- | --- | --- |
| Soler et al,2007 (28) | 131/142 | Outpatients aged 40 years, of either sex, with mild COPD or chronic bronchitis at the time of the adverse event | | Oral capsule | Alkaline bacterial lysate | One capsule daily for 30 days, followed by three 10-day courses at the start of months 3, 4, and 5, and then a one-month treatment-free period. | OM-85 (Broncho-Vaxom) |
|  |  |  |  |  |  |  | Placebo |
| Tang,et al,2015 ( 32) | (192/192) | Male or female patients aged 40–75 years with a ≥6-month physician-confirmed diagnosis of chronic bronchitis or COPD, experiencing a GOLD-defined acute exacerbation at enrollment, and an FEV₁ of 40–70% predicted within the prior 6 months | | Oral capsule | Alkaline bacterial lysate | Administration of the study drug or Placebo for 10 days each month | OM-85 |
|  |  |  |  |  |  |  | Placebo |
| Jing LI et al,2004 (24) | 41/49 | COPD patients presenting with chronic bronchitis | | Oral capsule | Alkaline bacterial lysate | One capsule daily during the first ten days of each month, administered consecutively for three months | OM-85 Broncho-Vaxom capsules |
|  |  |  |  |  |  |  |  |
|  |  |  |  |  |  |  | Placebo |
| Cazzola et al,2006 (33) | 86/92 | Patients diagnosed with moderate to very severe COPD GOLD II TO IV and able to understand instructions. | | Sublingual | Mechanical bacterial lysate | One treatment cycle per month for three months ,of daily sublingual administration of one PMBL tablet for 10 consecutive days per month,followed by a 12-month follow-up period | Ismigen |
|  |  |  |  |  |  |  | Placebo |
| Braido et al,2015 (9) | 142/146 | A confirmed diagnosis of COPD classified as moderate, severe, or very severe in accordance with GOLD criteria | | Sublingual | Mechanical bacterial lysate | Two treatment cycles of Ismigen, each lasting three months and consisting of daily sublingual administration of one PMBL tablet for 10 consecutive days per month, then 3 months off, after that the second cycle of therapy as described initiated , then 3 months without PMBL. | Ismigen |
|  |  |  |  |  |  |  | Placebo |
| Cazzola et al, 2009 (11) | 30/33 | Patients aged ≥50 years with moderate COPD and chronic bronchitis, undergoing regular treatment with salmeterol/fluticasone (SFC) 50/500 mg twice daily for moderate to very severe COPD | | Sublingual | Mechanical bacterial lysate | One tablet daily for the first ten days of each month, repeated consecutively for three months | Ismigen |
|  |  |  |  |  |  |  |  |
|  |  |  |  |  |  |  |  |
|  |  |  |  |  |  |  |  |
|  |  |  |  |  |  |  | Placebo |
| J.P. COLLET et al, 1997 (12) | 190/191 | Acute exacerbation of chronic obstructive pulmonary disease with FEV1 ranged between 20 to 70%, history of heavy smoking | | Oral capsule | Alkaline bacterial lysate | One capsule daily for the first ten days of each month, repeated for 12 months | OM-85 BV |
|  |  |  |  |  |  |  | Placebo |
| Avdeev et al, 2020 (20) | 30/30 | Patients with chronic obstructive pulmonary disease (COPD) who fall into GOLD categories C and D, reflecting a history of frequent exacerbations. | | Sublingual | Mechanical bacterial lysate | Each cycle involved daily sublingual administration of one tablet for 10 consecutive days, followed by 20 days of conventional therapy. This regimen was repeated monthly over a three-month period | Intervention **group** received conventional COPD therapy+ Mechanical Bacterial lysate(Ismigen ) |
|  |  |  |  |  |  |  | Control group , received conventional COPD therapy only (without Ismigen) |
| Menardo et al, 1985(25) | 21/23 | COPD patients with more than 3 infective exacerbations in preceding year | | Oral liquid | Bacterial Lysate | 10 ml orally for 20 days each month over 3 months | Diribiotine CK |
|  |  |  |  |  |  |  | Placebo |
| EUCTR2007 (23) | 171/169 | COPD patients | | Oral capsule | Alkaline bacterial Lysate | 7 mg daily for 1 month → no treatment for 1 month → 7 mg daily for first 10 days of 3 consecutive months | OM-85 |
|  |  |  |  |  |  |  | Placebo |
| Nishantha et al,2014. (26) | 21/24 | Clinically stable COPD patients with moderate to severe disease stage. | | Sublingual | Mechanical bacterial lysate | Sublingual tablet administered daily during the initial 10 days of three successive months | Ismigen |
|  |  |  |  |  |  |  | Placebo |
| Olivieri et al, 2011 (27) | 170/170 | Patients over 40 years old with GOLD stage II or III COPD, a documented history of at least two acute exacerbations in the past year, and an FEV₁ between 30% and 80% of predict | | Oral capsule | Alkaline bacterial lysate | 7mg oral capsule taken daily for 1 month, then stopped for 1 month, followed by 7 mg daily for the first 10 days of each of the next 3 months. | OM-85 |
|  |  |  |  |  |  |  | Placebo |
| Ricci et al,2014 (22) | 11/12 | Patients ≥40 years old diagnosed with moderate, severe, or very severe chronic obstructive pulmonary disease | | Sublingual | Mechanical bacterial lysate | 90-day treatment phase, participants received one of either PMBL or Placebo daily for the first 10 days of each 30-day interval. This was followed by a 90-day treatment-free period. | Ismigen |
|  |  |  |  |  |  |  | Placebo |
| Xinogalos et al,1993 (30) | 29/33 | COPD patients with 3 or more exacerbation during the previous year. | Oral capsule | | Alkaline bacterial Lysate | 7 mg daily for 1 month, no treatment for 1 month, then 7 mg daily for 10 consecutive days/month for 3 months (5 months | OM-85 |
|  |  |  |  |  |  |  | Placebo |
| Zeng et al. (2019) (31) | (72/78) | Participants with controlled COPD symptoms | | Oral capsule | Alkaline bacterial lysate | A daily capsule (OM-85, 7 mg) or Placebo administered for 10 consecutive days each month, over a period of 6 consecutive months. | OM-85 |
|  |  |  |  |  |  |  | Blank(conventional treatment) |
