# Supplementary material for: Evaluating the therapeutic benefit of bacterial lysates in chronic obstructive pulmonary disease: a systematic review and meta-analysis
Source: Front Med (Lausanne). 2026 Oct 21;13:1928291. doi: 10.3389/fmed.2026.1928291 (PMC13731251; doi:10.3389/fmed.2026.1928291)
Supplement: Supplementary file 2 [file Table2.docx]

| StudyID (Author/Year) | Total Participants(Placebo /treatment ) | Groups Compared | Age Range | Male (BL/  Placebo) | Smoking (N) BL/ Placebo) | FEV₁% (BL/ Placebo) | GOLD Severity (BL/ Placebo) | Flu vaccination | Long term effect bronchodilation |
| --- | --- | --- | --- | --- | --- | --- | --- | --- | --- |
| Soler,2007 (28) | (131/142) | OM-85 | 40-75 | 78 | 87/142 | 85 | (GOLD1) mild | Allowed | Allowed |
|  |  | Placebo | 40-75 | 57 | 77/131 | 82.6 | (GOLD1 )mild |  |  |
| Tang,2015 (32) | (192/192) | OM-85 | 40-75 | 141 | 123/192 | 54 | (GOLD2)moderate | Allowed | Allowed |
|  |  | Placebo | 40-75 | 133 | 120/192 | 55 | (GOLD2)moderate |  |  |
| Jing LI,2004 (24) | (41/49) | Broncho-Vaxom capsules | 63-71 | 27 | 49/49 | 60.9 | (GOLD2)moderate | NR | Allowed |
|  |  | Placebo | 60-70 | 22 | 41/41 | 67.2 | (GOLD2)moderate |  |  |
| Cazzola,2006 (33) | (86/92) | Ismigen | 64-68 | 60 | 41/92 | 46.2 | (GOLD3 )severe | NR | NR |
|  |  | Placebo | 64-70 | 59 | 37/86 | 45.7 | (GOLD3 )severe |  |  |
| Braido,2015 (9) | (142/146) | Ismigen | ≥40 | 107 | 123/146 | 52.18 | (GOLD2)moderate | Allowed | Allowed |
|  |  | Placebo | ≥40 | 91 | 120/142 | 52.66 | (GOLD2)moderate |  |  |
| Cazzola,2009 (11) | (30/33) | Ismigen | ≥50 | 25 | 16/33 | 47.7 | (GOLD3 )severe | Allowed | Allowed |
|  |  | Placebo | ≥50 | 26 | 19/30 | 46.2 | (GOLD3 )severe |  |  |
| J.P.COLLET 1997 (12) | (190/191) | OM-85 | NR | 133 | 78/191 | 67.7 | (GOLD2)moderate | NR | NR |
|  |  | Placebo | NR | 135 | 59/190 | 68.4 | (GOLD2)moderate |  |  |
| Avdeev, 2020(20) | (30/30) | Intervention group received conventional COPD therapy+ MechanicalBacterial (Ismigen) | 60.0-78.8 | 28 | NR | NR | (GOLD3- GOLD4)severe to very severe. | NR | NR |
|  |  | Control group received conventional COPD therapy only (without Ismigen) | 62.0-77.8 | 30 | NR | NR | (GOLD3- GOLD4) severe to very severe. | NR | NR |
| Menardo, 1985 (25) | (21/23) | Diribiotine CK | NR | 6 | 1/23 | NR | NR | NR | NR |
|  |  | Placebo | NR | 11 | 2/21 | NR | NR | NR | NR |
| Euctr,2007(23) | 171/169 | OM-85 | NR | NR | NR | ≥30% and ≤80% predicted | (GOLD2- GOLD 3)moderate to severe. | NR | NR |
|  |  | Placebo | NR | NR | NR | ≥30% and ≤80% predicted | (GOLD2-GOLD3)moderate to severe. | NR | NR |
| Nishantha, 2014 (26) | (21/24) | Ismigen | 50 -58  50 -58 | 24 | NR | NR | (GOLD3- GOLD4)severe to very severe. | NR | NR |
|  |  | Placebo |  | 21 | NR | NR | (GOLD3- GOLD4)severe to very sever. | NR | NR |
| Olivieri, 2011(27) | (170/170) | OM-85 | >40 | NR | NR | NR | (GOLD2- GOLD3)moderate to severe | NR | NR |
|  |  | Placebo | >40 | NR | NR | NR | (GOLD2- GOLD3)moderate to severe. | NR | NR |
| Ricci,2014 (22) | (11/12) | Ismigen | 65-89 | 14/22 | NR | NR | (GOLD2-3-4)moderate to severe to very severe. | NR | NR |
|  |  | Placebo | NR | 14/22 | NR | NR | (GOLD2- GOLD4)moderate to severe to very severe. | NR | NR |
| Xinogalos, 1993 (30) | (29/33) | OM-85 | 43.4 – 68.7 | 23 | 20 | NR | (GOLD2)moderate | NR | NR |
|  |  | Placebo | 46.9 – 72.6 | 20 | 16 | NR | (GOLD2)moderate | NR | NR |
| Zeng,2019 (31) | (72/78) | OM-85 | 64-75 | 67 | 63 | 49.15 | (GOLD3 )severe | NR | NR |
|  |  | Blank (conventional treatment) | 63-75 | 63 | 61 | 49.06 | (GOLD3 )severe | NR | NR |
